# Treatment of a Full-Thickness Burn Injury With NovoSorb Biodegradable Temporizing Matrix and RECELL Autologous Skin Cell Suspension: A Case Series

**DOI:** 10.1093/jbcr/irz179

**Published:** 2019-11-25

**Authors:** Kenneth W Larson, Cindy L Austin, Simon J Thompson

**Affiliations:** Trauma and Burn Research, Mercy Hospital – Springfield, Missouri

## Abstract

Dermal substitutes coupled with split thickness skin graft are the primary method of treating most severe full-thickness burns particularly when there is a lack of healthy donor skin. Although dermal replacements optimize functional and aesthetic outcomes in patients, the risk of infection and the amount of time required to process most dermal substitutes delay treatment potentially compromising graft take and the overall healing process. The purpose of this case series is to describe the treatment course of patients with severe burn injuries using a novel synthetic Biodegradable Temporizing Matrix (NovoSorb BTM) in conjunction with RECELL Autologous Cell Harvesting Device, a new methodology allowing for a timely point-of-care preparation of an autologous skin cell suspension in combination with a 3:1 split-thickness skin graft. To the best of our knowledge, this is the first reported case series to describe the treatment algorithm and clinical outcomes of deep full-thickness burns utilizing BTM in conjunction with RECELL ASCS.

Over the past 45 years, the process of skin tissue engineering has advanced bringing forth several cultivation methods of dermal substitutes to regenerate dermal and fascia tissue loss.^1,2^ The development of dermal substitutes in the management of burns has offered a variety of treatment options, both biological (autologous, allogeneic, and xenogenic) and synthetic (biodegradable and non-biodegradable).^3^ Despite the wide range of substitutes commercially available to treat large burns, each remains lacking in certain aspects. Deficiencies vary based on substitute type, yet common concerns include: 1) fragility of microstructure, 2) risk of infection, 3) risk of graft failure, 4) length of time to harvest/process and develop skin substitutes, and 5) high cost.^4^ This article describes three cases focusing on full-thickness (FT) burns combining two novel wound therapies: Novosorb Biodegradable Temporizing Matrix (BTM; Polynovo, Adelaide, Australia) in conjunction with RECELL Autologous Cell Harvesting Device (AVITA Medical, Valencia, California) for application of autologous skin cell suspension (ASCS) spray.

## CASE #1

**Figure 1.**
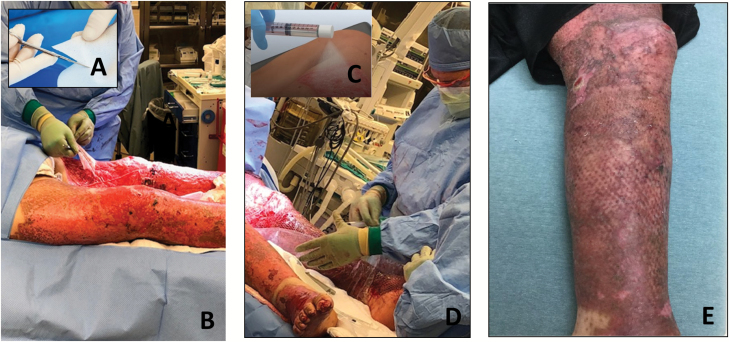
(A) NovoSorb Biodegradable Temporizing Matrix (BTM). (B) NovoSorb BTM delamination-left leg; (C) RECELL Autologous Skin Cell Suspension Spray Device; (D) RECELL ASCS sprayed over grafted leg; (E) Leg POD 69.

A 23-year-old male presented with 60% TBSA partial and FT thermal burns. On hospital day 4, burns to the left leg were tangentially excised down to the fascia. During the surgery, the patients’ temperature gradually decreased and he became slightly acidotic; therefore, in the best interest of the patient, the surgeon terminated the procedure and covered the leg with Mepitel AG (Molnlycke Healthcare, Gothenburg). On hospital day 5, the wound bed was re-prepped and BTM (Figure 1A) was placed to the left anterior thigh, posterior calf, and left foot. The BTM was used specifically for improving contour, appearance, and flexibility of the affected area because of the depth of the resection and to avoid grafting over muscle. On hospital day 10, the presence of an infection was noted; therefore, the patient underwent debridement and the BTM was replaced. Approximately 1 month after BTM placement and integration was achieved, the temporary seal layer of the BTM was removed (Figure 1B) and the dermal bed was prepared for split-thickness skin graft (STSG). Concurrently, donor skin was harvested at 0.008″ depth and then divided into 11 skin samples each measuring 2 × 3 cm. Using the RECELL System, these samples were placed into an enzyme solution to separate the cells. After 15 to 20 minutes in the enzymatic solution, the samples were scraped mechanically removing the epidermis from the dermis with attention to the epidermal-dermal junction. A 3:1 meshed STSG was secured to the wound bed and ASCS was applied.(Figure 1C and D) The cell suspension was sprayed on the leg from the top to down to minimize run-off waste. The wound was dressed with Telfa Clear Wound Dressing (Covidien, Minneapolis, MN), a nonadherent, nonabsorbent, small pore primary dressing, and Xeroform Occlusive Petrolatum Gauze (Covidien). A protective outer gauze dressing was applied. Standard protocol indicates using one layer of Telfa Clear. Notably, per the discretion of the surgical team, in certain areas two layers of Telfa Clear were placed to overlap areas prone to shearing forces without disturbing the wound composition and to minimize the amount of friction.

Five days after surgery, range of motion was assessed, and the patient had left knee flexion to ~30°. At 1-week postautografting, the leg was 90% re-epithelialized and by 2 weeks, the leg was considered healed (>95% re-epithelialization). At postop day (POD) 10, the patient was able to walk 25 feet with moderate assistance, and at POD 15 the patient had knee flexion to 65°. By POD 26, 100° knee flexion obtained, and the newly formed skin remained robust and durable. On POD 35, the patient was discharged from physical/occupational rehab. However, the patient was noncompliant in completing home rehabilitation exercises; therefore, a decline in progress was noted. At POD 47, the patient had 82° knee flexion. Despite the delay in progress by POD 69, the patient had knee flexion 95° and maintained positive outcomes (Figure 1E). At 4 months, postop assessment was reported as mildly mismatched in color, pigment, and texture. See Table 1 for Vancouver Scar Scale (VSS) assessment of cases.

**Table 1.** Vancouver Scar Scale^29^

	Case 1	Case 2			Case 3	
	Leg	Arm	Torso	Thigh	Torso	BUEs
	POD 69	POD 30	POD 30	POD 30	POD 34	POD 51
Pigmentation	hypo	hyper	hyper	hyper	hyper	hyper
Vascularity	purple	pink-red	pink-red	red	red-purple	red-purple
Pliability	yielding	supple	yielding	firm	contracture	contracture
Height	3 mm	<2 mm	<2 mm	<2 mm	<2 mm	<5 mm

## CASE #2

**Figure 2.**
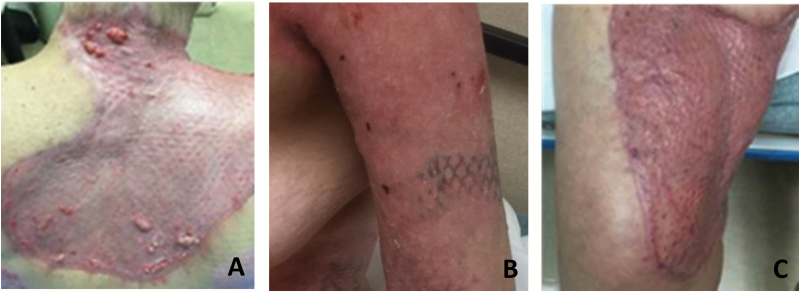
(A) Posterior torso POD 21. (B) Left upper arm POD 29. (C) Thigh POD 29.

The patient is a 53-year-old female who presented with 35% TBSA thermal burns. On hospital day 3, she received tangential excision of FT burns dressed with antibiotic ointment, Xeroform, and gauze. On hospital day 5, the right thigh and left upper arm were excised down to fascia and covered in BTM and Mepitel AG (Molnlycke, Gothenburg, Sweden) along with tangential excision to the upper posterior torso with placement of BTM. After 28 days, the patient underwent surgery for touch up grafting, excisional debridement of tissue including removal of the temporal layer of BTM. The dermal bed was prepared for the placement of 4:1 meshed STSG and ASCS spray prepared with RECELL and covered with Telfa Clear, Xeroform, gauze, and wraps. Using 4:1 meshed STSG resulted in less donor site (DS) skin needed to achieve burn coverage. The majority of grafts healed, upper torso noted with presence of some hypergranulation tissue (Figure 2). Overall, this patient resulted in acceptable healing with no occurrence of wound infection.

## CASE #3

**Figure 3.**
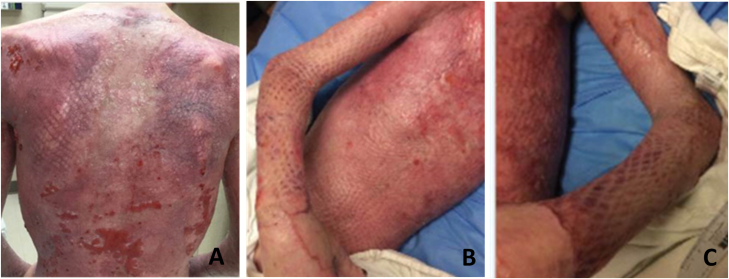
(A) Posterior torso POD 35. (B) Right arm POD 51. (C) Left arm POD 51.

The patient is a 21-year-old male who presented with 60% TBSA, the majority being FT thermal burns to the posterior and anterior torso, and bilateral upper extremities (BUE). On hospital day 2, the patient underwent tangential excision down to fascia of the BUEs with placement of BTM; however, during the procedure the patient became unstable and surgery was halted. Four days later, the torso was excised and BTM was placed. Once the BTM was adequately vascularized (approximately 1 mo later), the patient returned to surgery for a 3:1 meshed STSG in combination with the application of ASCS spray. On the posterior torso, the patient developed a Pseudomonas like drainage/odor; therefore, application of the STSG and ASCS spray was postponed. Notably, BTM was not lost and persisted in the presence of infection. Once the signs or symptoms of infection cleared and adequate donor skin became available the patient underwent graft surgery coupled with ASCS spray. Overall, the patient recovered well with acceptable outcomes. (Figure 3)

## DISCUSSION

Deep burns particularly of the lower extremities present a treatment challenge to burn care professionals due to many cases of exposed muscles, tendons, bones, and joints after debridement.^5^ In such severe cases, conventional treatment would include the placement of a dermal substitute or a Cultured Epidermal Autograft (CEA) in conjunction with a STSG. More recently a novel method applying ASCS in combination with a STSG is available.

Traditionally, Integra Dermal Regenerative Template has been suggested to be the “gold standard” dermal substitute for FT burns that extend through the dermis.^4,6,7^ However, reluctance to use Integra has been noted due to risk of infection, early spontaneous delamination, graft take failure, thicker scarring, wound contraction and relatively high cost.^8,9^ In this case series, surgeons elected to use NovoSorb Biodegradable Temporizing Matrix as an intermediate protective covering.

### Biodegradable Temporizing Matrix

Comparatively, BTM offers a thicker, more durable reconstruction framework which is biodegradable and biocompatible^10^ and is considerably less costly and more robust to infection.^8^ Currently BTM is the only synthetic, nonbiological, polyurethane temporal dermal substitute intended for use in replacement of tissue loss.^10^ BTM is composed of three layers including biodegrading foam, bonding layer, and sealing membrane which acts as an interface for deep burns down to the muscle. BTM serves as a 3D scaffold for structural support allowing blood vessels and fibroblasts to infiltrate and then proliferate over 3 to 4 weeks. The dermal matrix prevents wound contraction while allowing re-epithelialization.^8,11^ Once the tissue is vascularized, the seal is peeled away, and the wound surface is reconstructed and ready for a permanent cover.^10^ BTM has been clinically proven in patient studies to demonstrate no signs of infection^8,12^ as well as persist in the presence of infection while sustaining STSG.^13^ Recent preliminary results (2019) evaluating 36 patient cases suggests that BTM offers a higher resistance to infection and proved to be a reliable method and stable platform in resurfacing acute burns and chronic wounds.^14^

### Cultured Epidermal Autografts

The use of CEAs has been used in the treatment of large burns since the early 1980s.^15^ CEAs involve growing out the patient’s keratinocytes in a culture to form a confluent epithelial sheet cover for grafting. These sheets are fragile and highly susceptible to infection after placement.^16,17^ Recently, a 25-year review of CEAs in large burns (mean TBSA 67% (±17) reported infections as the most frequent adverse reaction.^18^ One of the major shortcomings of CEAs is the lengthy production time. The development process can take between 12 days and 4 weeks to prepare with only a short 2–3 day window for the sheet to take.^19,20^ This short window alludes to another challenge; the time delay between harvest and the unpredictability of when the lab cultured dermal sheet will be ready for placement.^21^ Given the fragile nature, calculating the most optimal time for surgery can be tricky since the readiness of the wound bed needs to coincide with the availability of the prepared dermal substitute.^4,16,21^ A 10-year literature review evaluating CEAs in severe burn patients reported an average clinical graft take of only 45%, and over half of all the cells cultured had to be discarded due to complications involved in timing the production of CEA sheets to meet the needs of the patients.^19^ A more recent examination (2019) including 954 patients with a mean TBSA 67% (±17) reported an average graft take at discharge with CEAs as 75%.^18^ Additional concerns of CEA’s include blistering, shearing, itching, graft loss, requirement of repeat coverage, and wound contracture.^16^ Interestingly, James et al demonstrated a reduction in healing time by virtue of spraying cultured keratinocytes as opposed to preparing the cultured keratinocytes into sheets.^22^

### RECELL ASCS

After BTM application, the surgeons elected to use a novel cell harvesting system RECELL to aid in regeneration of a permanent outer layer of skin. An FDA-approved spray application methodology using an ASCS derived from noncultured skin cells. A small sample of the patient’s skin is processed using the RECELL System to achieve a spray-on skin suspension that contains keratinocytes, fibroblasts, and melanocytes. Each 1-cm^2^ biopsy can cover up to an 80-cm^2^ area. The cell suspension is processed in the operating room and can be ready within 30 minutes for application in combination with meshed autografts on FT or alone on deep partial-thickness burns. This technology in deep partial thickness burns has been shown to minimize DS size and morbidity, eliminate the need for costly and time-consuming laboratory culture, and reduce hospital cost.^16,23–26^ This technology in the treatment of FT burns has shown that re-epithelization can be achieved using a much smaller DS compared with traditional autografting.^27^ Holmes et al reported that widely meshed STSG in combination with RECELL ASCS demonstrated a significant (*P* < .001) reduction (32%) in donor skin requirements with improved healing, pain, and scarring compared with traditional grafting.^27^ Recently Kowal et al evaluated cost and resource use from three U.S. burn centers. The ASCS treatment resulted in a shorter length of stay, saving 14% to 17% yearly, compared with standard of care. Similarly, Foster et al projected calculations comparing the ASCS system vs standard of care could save 16% total costs in a year, with the largest cost saving reduction due to length of stay, with approximately 67% less autografting procedures, and a further 13% decrease in operating room time.^28^

## CONCLUSIONS

Ideally, a dermal substitute has tissue compatibility, is relatively cost effective, readily available, and has a low risk of antigenicity and disease transmission. Indeed, given the size of the burns, the surgeons elected to use RECELL with 3:1 and 4:1 graft vs a traditional 2:1 graft, thus requiring less DS skin needed for burn site coverage. Furthermore, as reported in the literature, one case demonstrated that BTM can persist in the presence of an infection and successfully sustain integration while treating the patient systemically thus not extending the length of stay. Together, the use of the BTM and RECELL ASCS appears to alleviate some of the current challenges of dermal substitutes. These novel cases demonstrated that BTM used in conjunction with RECELL ASCS successfully achieved definitive closure of FT burn wounds and demonstrated acceptable outcomes.

## LIMITATIONS

Potential limitation of this case report include bias in rating wound healing and time to closure. Time and cost in operating room was not calculated and it is unknown if a 3:1 graft alone would have produced the same outcome. Pain was not evaluated.
